# The role of SHR in risk stratification for long-term prognosis in patients with coronary artery disease: findings from a large cohort study

**DOI:** 10.3389/fendo.2025.1640725

**Published:** 2025-09-16

**Authors:** Wei Xu, Xiang Wang, Chenxi Xia, Xuyang Meng, Yi Li, Yejing Zhao, Chenguang Yang, Baoyu Feng, Zinan Zhao, Fang Wang

**Affiliations:** ^1^ Department of Cardiology, Fuwai Hospital, National Center for Cardiovascular Diseases, State Key Laboratory of Cardiovascular Disease, Chinese Academy of Medical Sciences and Peking Union Medical College, Beijing, China; ^2^ Department of Cardiology, Beijing Hospital, National Center of Gerontology, Institute of Geriatric Medicine, Chinese Academy of Medical Sciences, Beijing, China; ^3^ Peking University Fifth School of Clinical Medicine, Beijing, China; ^4^ Graduate School of Peking Union Medical College, Chinese Academy of Medical Sciences, Beijing, China; ^5^ Department of Gerontology, Beijing Hospital, National Center of Gerontology, Institute of Geriatric Medicine, Chinese Academy of Medical Sciences, Beijing, China; ^6^ Department of Clinical Trial Center, Beijing Tiantan Hospital, Capital Medical University, Beijing, China; ^7^ Department of Pharmacy, Beijing Hospital, National Center of Gerontology, Institute of Geriatric Medicine, Chinese Academy of Medical Sciences, Beijing Key Laboratory of Assessment of Clinical Drugs Risk and Individual Application (Beijing Hospital), Beijing, China

**Keywords:** coronary artery disease, stress hyperglycemia ratio, all-cause death, cardiovascular disease death, major adverse cardiovascular events

## Abstract

**Background:**

Recently, the Stress Hyperglycemia Ratio (SHR) — which integrates acute increases in blood glucose with long-term glycemic control levels — has shown independent predictive value for adverse events in patients with acute coronary syndrome (ACS). However, the long-term prognostic significance of SHR in a broader population of coronary artery disease (CAD) remains unclear. This study aimed to explore the role of SHR in prediction of long-term prognosis of CAD.

**Methods:**

In this cohort study, we enrolled 23,591 participants diagnosed with CAD from January, 2016, to December, 2021 in Beijing Hospital. After excluding patients lacking data, with cancers, or missing follow-ups, 7,162 patients were finally enrolled into the analyses. The SHR was calculated using the following equation: SHR = admission glucose (mmol/L)/(1.59 × HbA1c [%]-2.59). The 7,162 participants were divided into three groups based on SHR tertiles: Tertile 1 (SHR ≤ 0.72, n=2391), Tertile 2 (0.73≤SHR ≤ 0.82, n=2388), and Tertile 3 group (SHR≥0.83, n=2383). The primary endpoint was all-cause mortality and cardiovascular death (CVD), while the second endpoint was major adverse cardiovascular events (MACE). The median follow-up was 28 months.

**Results:**

Our results suggest that SHR was significantly associated with increased risks of long-term all-cause death, CVD death, and MACE. The Kaplan-Meier curves revealed that the highest tertile (T3) group had the highest risk of all-cause death, CVD death, and MACE, while the lowest tertile (T1) group had the lowest risk (all log-rank P < 0.05). After adjusting risk factors, the results of cox regression analyses showed that SHR was significantly associated with all three outcomes (all P < 0.05). For all-cause death, SHR was associated with an increased risk of all-cause death in the fully adjusted model (Model 3: HR = 2.52, 95% CI: 1.57 – 4.05, P < 0.001). Compared to the lowest tertile (T1), participants in the highest tertile (T3) had a likely higher risk of all-cause death (HR = 1.40, 95% CI: 1.05 – 1.87, P = 0.021). SHR also demonstrated a positive association with CVD death (Model 3: HR = 2.87, 95% CI: 1.22 – 6.76, P = 0.016), and participants in T3 had a significantly higher risk of CVD death compared to T1 (HR = 1.94, 95% CI: 1.11 – 3.40, P = 0.021). Additionally, SHR was also independently associated with MACE (Model 3: HR = 1.70, 95% CI: 1.21 – 2.38, P = 0.002). The risk of MACE was significantly higher in T3 compared to T1 (HR = 1.21, 95% CI: 1.02 – 1.45, P = 0.031). The restricted cubic spline (RCS) analysis further confirmed a positive nonlinear association between SHR and these adverse outcomes (all-cause death, CVD death, and MACE) and exhibited a J-shaped curve.

**Conclusions:**

SHR is significantly associated with long-term all-cause death, CVD death, and MACE in CAD patients. Our findings highlight SHR can be used as a valuable tool for long-term prognosis risk stratification in CAD, potentially influencing clinical decision-making and patient management strategies.

## 1 Introduction

Coronary artery disease (CAD) is a leading cause of morbidity and mortality worldwide (1). The progression of CAD is dynamic and may lead to major adverse cardiovascular events (MACE), death or cardiovascular death (CVD). The early identification of high-risk populations for adverse outcomes in CAD remains a key strategy to optimize CAD management. Stress hyperglycemia, defined as transient hyperglycemia occurring in response to acute physiological stress, has been considered a predictor for adverse events, especially in patients with emergency and critical illness (2, 3). To distinguish from individuals with poor chronic glycemic control, Robert et al. proposed the stress hyperglycemia ratio (SHR), which is calculated as the admission blood glucose (ABG) divided by the estimated average glucose derived from HbA1c, to serve as a more accurate indicator of acute glycemic dysregulation (4). In recent years, SHR has been reported to be associated with increased risks of MACE and mortality in patients with ACS and chronic coronary syndromes (CCS) (5, 6). However, conflicting findings exist regarding the prognostic value of SHR across different CAD subtypes and glycemic statuses. Thus, further investigation is warranted to elucidate the role of SHR in predicting long-term cardiovascular outcomes among patients with CAD. This study serves as a natural extension of our previous research (6). In our prior investigation, we examined the correlation between the SHR and in-hospital mortality within the identical cohort. Our findings revealed that SHR exhibited a significant association with in-hospital mortality among patients with ACS or CCS, particularly among those with prediabetes and diabetes mellitus. Upon completion of the follow-up period, the current study builds upon and expands the insights garnered from our initial exploration, delving deeper into the associations between SHR and long-term prognosis.

The aim of this study was to evaluate the association between SHR and long-term all-cause death, CVD, and MACE in a large cohort of CAD patients.

## 2 Methods

### 2.1 Study design and population

**Figure 1:**
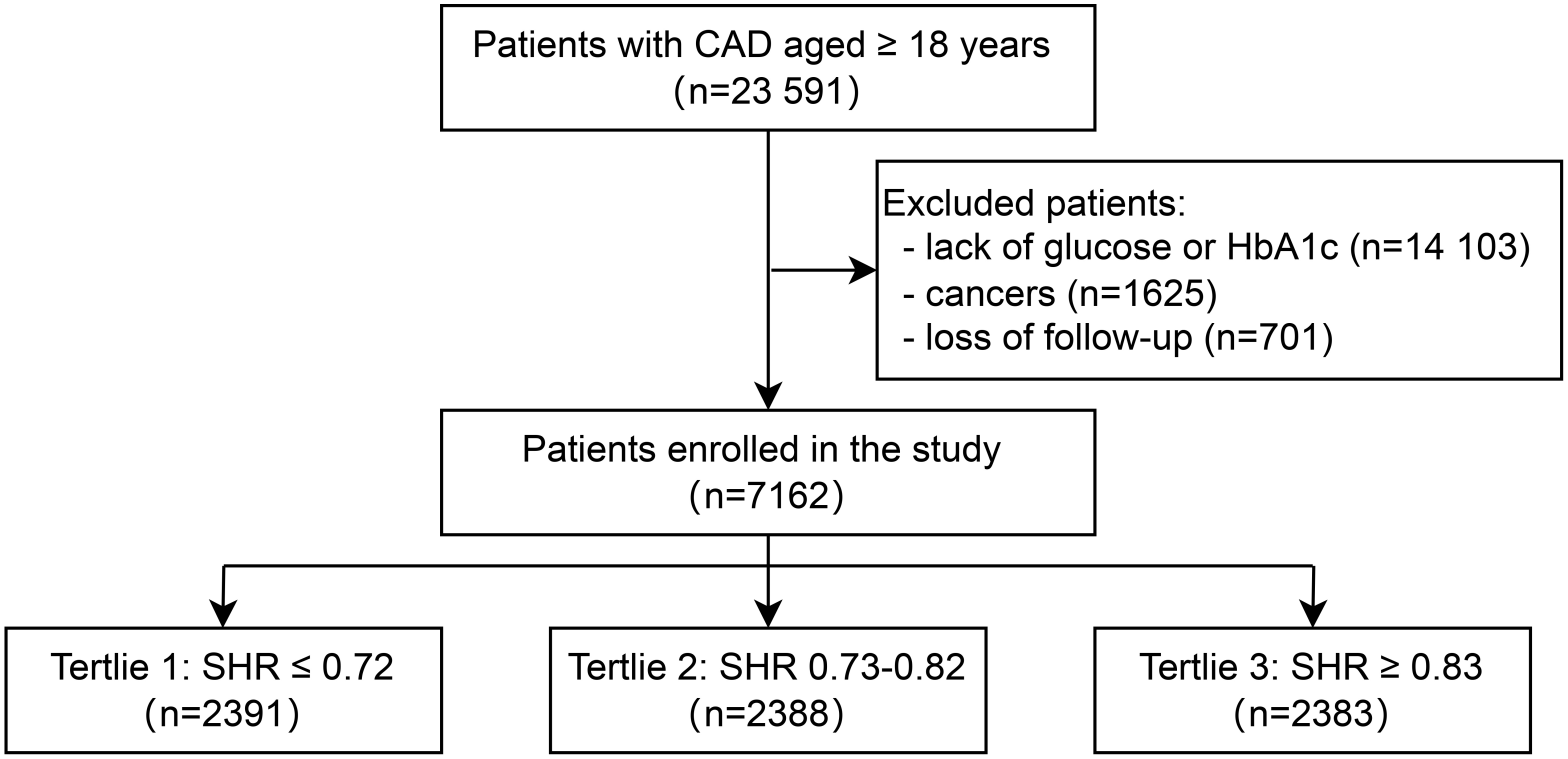
Flowchart of this study.

This study was conducted at Beijing Hospital from January 2016 to December 2021. A total of 23,591 patients diagnosed with CAD were enrolled. All CAD patients were from the inpatient department of Beijing Hospital and had at least one major coronary artery stenosis ≥50% diagnosed by coronary CT or angiography. After excluding patients with missing blood glucose or HbA1c data (n=14,103), those diagnosed with cancer (n=1,625), and those who were lost to follow-up (n=701), a total of 7,162 patients were included in the final analysis (**Figure 1**). Based on the tertiles of the SHR, the 7,162 patients were divided into three groups: Tertile 1 (SHR ≤ 0.72, n=2,391), Tertile 2 (0.73≤SHR ≤ 0.82, n=2,388), and Tertile 3 (SHR≥0.83, n=2,383). The median follow-up duration was 28 months. The primary outcomes of this study were all-cause death and CVD. The secondary outcome was MACE. The study was approved by the Ethics Committee of Beijing Hospital and conducted in accordance with the Declaration of Helsinki.

### 2.2 Data collection and measurements

The SHR was calculated as the ABG (mmol/L) divided by the estimated average glucose (eAG), where eAG was determined using the formula: eAG = 1.59 × HbA1c [%] − 2.59 (4). The formula of eAG (mmol/l) was derived from the eAG (mg/dL) = 28.7 × HbA1c − 46.7, which was proposed by Nathan et al (7). We finally adopted the mmol/L version to match our laboratory units and ensure consistency between numerator and denominator in the SHR calculation. Baseline characteristics, including demographics (age, gender, height, weight, smoking status, and alcohol use) and vital signs (admission systolic blood pressure [SBP], diastolic blood pressure [DBP], and heart rate [HR]), were collected from medical records. Body mass index (BMI) was calculated as weight (kg) divided by height squared (m²). HbA1c, total cholesterol (TC), triglycerides (TG), high-density lipoprotein cholesterol (HDL-C), and low-density lipoprotein cholesterol (LDL-c) were measured from a cubital vein blood sample after at least eight hours of fasting. Blood glucose, TC, TG, HDL-C, and LDL-C were analyzed using a LABOSPECT 008 system (Hitachi, Tokyo, Japan), while HbA1c levels were determined by high-performance liquid chromatography (G8, TOSOH, Tokyo, Japan) at the laboratory of Beijing Hospital. The estimated glomerular filtration rate (eGFR) was calculated using the Chronic Kidney Disease Epidemiology Collaboration (CKD-EPI) creatinine equation.

### 2.3 Definitions

In this study, diabetes specifically refers to type 2 diabetes, which was defined as either a documented history of type 2 diabetes or an HbA1c level greater than 6.5% (14). Prediabetes mellitus (Pre-DM) was identified in patients who had never been diagnosed with diabetes but had an HbA1c level ranging from 5.7% to 6.4%. Normoglycemia (NGR) was assigned to those without a history of diabetes and with an HbA1c level of 5.7% or lower.

All-cause mortality included in-hospital deaths and deaths occurring during the follow-up period. CVD death in this study was defined as death resulting from cardiovascular diseases causes. MACE included rehospitalization due to cardiovascular causes or cerebrovascular causes, the occurrence of heart failure or cerebrovascular events (ischemic stroke or intracerebral hemorrhage) during follow-up, all-cause death, and CVD death.

### 2.4 Statistical analysis

Continuous variables were summarized as means ± standard deviations (SD) or medians with interquartile ranges (IQR), and categorical variables were counts and proportions. Differences for baseline characteristics across SHR tertiles were compared using one-way analysis of variance (ANOVA) or the Kruskal-Wallis test for continuous variables, and the chi-square test for categorical variables. Survival curves were plot by Kaplan-Meier method, and differences among groups were compared by log-rank test. Cox proportional hazards regression models were used to evaluate the association of SHR with all-cause death, death caused by CVD, and MACE. SHR was analyzed both as a continuous variable and as tertiles (T1 ≤ 0.72, T2 0.73 - 0.82, and T3 ≥ 0.83). Univariate and multivariable adjusted HRs were assessed based on three models: Model 1 was unadjusted; Model 2 was adjusted for age and sex; and Model 3 was further adjusted for potential confounders, including BMI, SBP, smoking, drinking, ACS, TC, LDL-c, eGFR, and HR. Restricted cubic spline (RCS) regression was used to explore the relationship between SHR and the risk of all-cause death, CVD, and MACE. Subgroup analyses were further performed to explore the association of SHR with outcomes stratified by ACS status (ACS vs. CCS) and glucose status (NGR, Pre-DM, and DM). All analyses were conducted using SAS 9.4. A two-tailed P value < 0.05 was considered statistically significant.

## 3 Results

### 3.1 Baseline characteristics of participants

Patients with CAD aged ≥ 18 years were included (N = 23,591). After patients lack of glucose or HbA1c (N = 14,103), with cancers (N = 1625), and loss of follow-up (N = 701) were excluded, a total of 7162 participants were included (**Figure 1**). There were 2391, 2388, and 2383 participants in the T1 group, T2 group, and T3 group. The baseline characteristics of participants stratified by SHR tertiles (T1 ≤ 0.72, T2 0.73 – 0.82, T3 ≥ 0.83) are presented in **Table 1**. Participants in the highest tertile (T3) were younger, had a higher proportion of men, and were more likely to smoke and drink. Besides, participants in T3 had higher BMI, TC levels, and ABG. The use of antidiabetic drugs was more prevalent in T1, while hypolipidemic drug use was higher in T2. No significant differences were observed in SBP or hypertension prevalence across tertiles.

**Table 1:** Baseline characteristics.

Variables	Total	T1 (≤0.72)	T2 (0.73 - 0.82)	T3 (≥0.83)	P Value
N	7162	2391	2388	2383	
Age, years	67.5 ± 10.9	68.9 ± 10.3	66.6 ± 10.8	66.8 ± 11.4	<0.001
Men, %	3089 (43.1)	969 (40.5)	1009 (42.3)	1111 (46.6)	<0.001
Smoking, %	3089 (43.1)	969 (40.5)	1009 (42.3)	1111 (46.6)	<0.001
Drinking, %	4126 (57.6)	1282 (53.6)	1376 (57.6)	1468 (61.6)	<0.001
BMI, kg/m^2^	25.6 ± 3.5	25.5 ± 3.5	25.6 ± 3.4	25.9 ± 3.5	<0.001
SBP, mmHg	136.3 ± 19.1	136.0 ± 18.6	135.8 ± 18.3	136.9 ± 20.3	0.086
DBP, mmHg	76.8 ± 12.1	76.0 ± 11.8	77.3 ± 12.0	77.2 ± 12.5	0.002
TC, mmol/L	3.8 ± 1.0	3.7 ± 1.0	3.9 ± 1.0	3.9 ± 1.0	<0.001
TG, mmol/L	1.2 (0.9 – 1.7)	1.1 (0.8 – 1.6)	1.2 (0.9 – 1.7)	1.3 (0.9 – 1.9)	<0.001
LDL-c, mmol/L	2.2 ± 0.8	2.1 ± 0.8	2.2 ± 0.9	2.2 ± 0.8	<0.001
HDL-c, mmol/L	1.1 ± 0.3	1.1 ± 0.3	1.1 ± 0.3	1.0 ± 0.3	<0.001
HbA1c, %	6.8 ± 1.4	7.2 ± 1.5	6.4 ± 1.0	6.7 ± 1.4	<0.001
ABG, mmol/L	6.5 ± 2.5	5.5 ± 1.3	5.9 ± 1.3	8.2 ± 3.3	<0.001
eGFR, mL/min/1.73m^2^	88.2 (74.0 – 96.5)	86.8 (71.4 – 95.3)	89.3 (77.9 – 96.7)	88.4 (72.4 – 97.5)	<0.001
Antidiabetic drugs, %	2709 (37.8)	1060 (44.3)	678 (28.4)	971 (40.7)	<0.001
Antihypertensive drugs, %	5481 (76.5)	1853 (77.5)	1818 (76.1)	1810 (76.0)	0.387
Hypolipidemic drugs, %	6087 (85.0)	2044 (85.5)	2071 (86.7)	1972 (82.8)	<0.001
Antiplatelet drugs, %	5744 (80.2)	1942 (81.2)	1947 (81.5)	1855 (77.8)	0.002
Hypertension, %	6488 (90.6)	2173 (90.9)	2143 (89.7)	2172 (91.1)	0.210
Glucose status, %					<0.001
NGR	897 (12.5)	78 (3.3)	358 (15.0)	461 (19.3)	<0.001
Pre-DM	2282 (31.9)	739 (30.9)	990 (41.5)	553 (23.2)	<0.001
DM	3983 (55.6)	1574 (65.8)	1040 (43.6)	1369 (57.4)	<0.001
Dyslipidemia, %	6716 (93.8)	2247 (94.0)	2253 (94.3)	2216 (93.0)	0.135
HR, bpm	77.6 ± 13.5	76.5 ± 13.0	77.0 ± 13.2	79.2 ± 14.1	<0.001
ACS, %	2711 (37.9)	942 (39.4)	944 (39.5)	825 (34.6)	<0.001
SHR	0.8 ± 0.2	0.6 ± 0.1	0.8 ± 0.1	1.0 ± 0.2	<0.001

Data are means ± standard deviation, numbers (%), or medians (interquartile range).

BMI, body mass index; SBP, systolic blood pressure; DBP, diastolic blood pressure; TG, triglyceride; TC, total cholesterol; LDL-c, low-density lipoprotein cholesterol; HDL-C, high-density lipoprotein cholesterol; ABG, admission blood glucose; eGFR, estimated glomerular filtration rate; HR, heart rate; ACS, acute coronary syndrome; SHR, stress hyperglycemia ratio; NGR, Normoglycemia; DM, diabetes mellitus.

**Figure 2:**
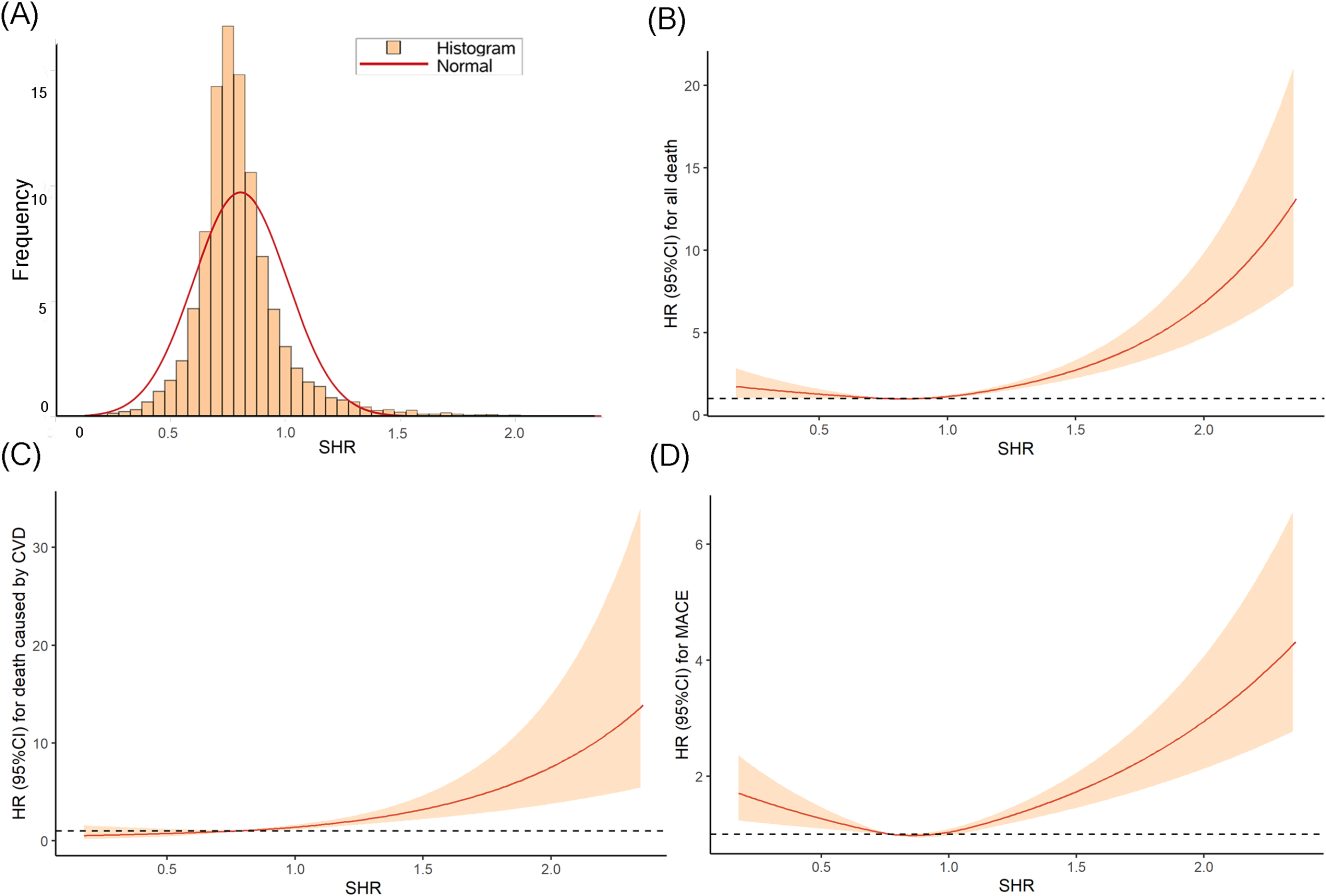
The distribution of SHR **(A)** and the RCS curves between SHR and all-cause death **(B)**, CVD death **(C)**, and MACE **(D)**.

The distribution of SHR approximated a normal distribution (**Figure 2A**), with a mean ± SD of 0.8 ± 0.2. The means ± SDs for the T1, T2 and T3 group were 0.6 ± 0.1, 0.8 ± 0.1, and 1.0 ± 0.2, respectively.

### 3.2 Association of SHR with all-cause death, CVD death, and MACE

**Figure 3:**
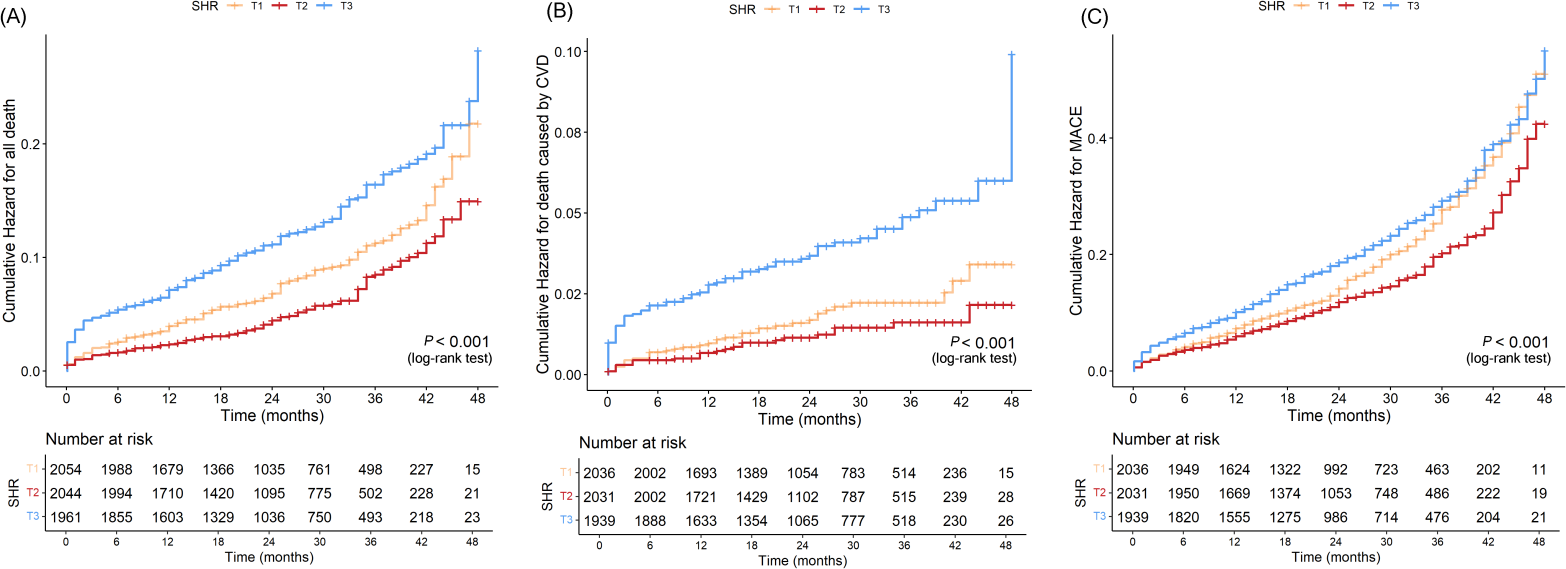
Kaplan–Meier curves for SHR and all-cause death **(A)**, CVD death **(B)**, and MACE **(C)**.

The Kaplan-Meier curves (**Figure 3**) revealed that the highest tertile (T3) group had the highest risk of all-cause death, CVD death, and MACE, while the lowest tertile (T1) group had the lowest risk (all log-rank P < 0.05). The association between SHR and all-cause death, death caused by CVD, and MACE, was shown in **Table 2**. After adjusting risk factors including age, sex, BMI, SBP, smoking, drinking, ACS, TC, LDL-c, eGFR, and HR in model 3, the results of cox regression analyses showed that SHR was significantly associated with all three outcomes (all P < 0.05). For all-cause death, SHR was associated with an increased risk of all-cause death in the fully adjusted model (Model 3: HR = 2.52, 95% CI: 1.57 – 4.05, P < 0.001). Compared to the lowest tertile (T1), participants in the highest tertile (T3) had a likely higher risk of all-cause death (HR = 1.40, 95% CI: 1.05 – 1.87, P = 0.021). SHR also demonstrated a positive association with CVD death (Model 3: HR = 2.87, 95% CI: 1.22 – 6.67, P = 0.016), and participants in T3 had a significantly higher risk of CVD death compared to T1 (HR = 1.94, 95% CI: 1.11 – 3.40, P = 0.021). Additionally, SHR was also independently associated with MACE (Model 3: HR = 1.70, 95% CI: 1.21 – 2.38, P = 0.002). The risk of MACE was significantly higher in T3 compared to T1 (HR = 1.21, 95% CI: 1.02 – 1.45, P = 0.031). Furthermore, the RCS curves showed a J-shaped positive association between SHR and all-cause death, CVD death, and MACE (**Figures 2B–D**).

**Table 2:** Association of SHR with all death, CVD death, and MACE.

	Model 1		Model 2			Model 3	
	HR (95%CI)	P value	HR (95%CI)	P value		HR (95%CI)	P value
All death							
SHR	3.14 (2.31 - 4.25)	<0.001	3.01 (2.23 - 4.07)	<0.001		2.52 (1.57 - 4.05)	<0.001
T1							
T2	0.72 (0.57 - 0.91)	0.006	0.85 (0.67 - 1.07)	0.172		0.90 (0.66 - 1.25)	0.541
T3	1.39 (1.14 - 1.69)	0.001	1.56 (1.28 - 1.90)	<0.001		1.40 (1.05 - 1.87)	0.021
CVD death							
SHR	4.86 (2.91 - 8.11)	<0.001	4.31 (2.58 - 7.18)	<0.001		2.87 (1.22 - 6.76)	0.016
T1	1						
T2	0.67 (0.40 - 1.11)	0.121	0.78 (0.47 - 1.30)	0.338		0.88 (0.45 - 1.73)	0.714
T3	2.06 (1.40 - 3.05)	<0.001	2.27 (1.53 - 3.36)	<0.001		1.94 (1.11 - 3.40)	0.021
MACE							
SHR	1.79 (1.40 - 2.29)	<0.001	1.81 (1.43 - 2.29)	<0.001		1.70 (1.21 - 2.38)	0.002
T1							
T2	0.81 (0.70 - 0.93)	0.003	0.89 (0.77 - 1.03)	0.117		0.98 (0.82 - 1.18)	0.856
T3	1.12 (0.99 - 1.28)	0.080	1.23 (1.08 - 1.41)	0.002		1.21 (1.02 - 1.45)	0.031

CVD, cardiovascular disease; MACE, major adverse cardiovascular events; HR,hazard ratio; SHR, stress hyperglycemia ratio.

Model 1: univariate model; Model 2: adjust for age, and sex; Model 3: adjust for age, sex, BMI, SBP, smoking, drinking, ACS, TC, LDL-c, eGFR, and HR.

### 3.3 Subgroup analysis

In subgroup analysis of ACS and chronic coronary syndrome (CCS), SHR was significantly associated with all-cause death, CVD death, and MACE in participants with CCS, but showed weaker associations in those with ACS (see **Table 3**).

**Table 3:** Association of SHR with all-cause death, CVD death, and MACE by ACS and CCS.

	Model 1		Model 2		Model 3	
	HR (95%CI)	P value	HR (95%CI)	P value	HR (95%CI)	P value
All death						
ACS						
SHR	2.39 (1.24 - 4.61)	0.009	2.94 (1.56 - 5.52)	0.001	2.09 (0.96 - 4.55)	0.064
T1	Ref.		Ref.		Ref.	
T2	0.67 (0.45 - 0.98)	0.038	0.74 (0.51 - 1.09)	0.124	0.81 (0.51 - 1.28)	0.356
T3	1.18 (0.84 - 1.65)	0.344	1.42 (1.01 - 1.99)	0.044	1.22 (0.79 - 1.86)	0.369
CCS						
SHR	3.32 (2.35 - 4.69)	<0.001	3.05 (2.16 - 4.31)	<0.001	2.98 (1.63 - 5.45)	<0.001
T1	Ref.		Ref.		Ref.	
T2	0.76 (0.57 - 1.02)	0.067	0.92 (0.69 - 1.24)	0.592	0.99 (0.63 - 1.56)	0.967
T3	1.50 (1.18 - 1.92)	0.001	1.66 (1.30 - 2.12)	<0.001	1.55 (1.04 - 2.30)	0.031
CVD death						
ACS						
SHR	3.33 (1.06 - 10.44)	0.039	3.58 (1.19 - 10.78)	0.024	1.53 (0.34 - 6.80)	0.577
T1	Ref.		Ref.		Ref.	
T2	0.49 (0.21 - 1.15)	0.103	0.54 (0.23 - 1.27)	0.161	0.73 (0.29 - 1.80)	0.492
T3	1.80 (0.97 - 3.35)	0.065	2.04 (1.09 - 3.80)	0.025	1.47 (0.68 - 3.17)	0.327
**CCS**						
SHR	5.42 (3.05 - 9.64)	<0.001	4.60 (2.57 - 8.23)	<0.001	5.29 (1.82 - 15.38)	0.002
T1	Ref.		Ref.		Ref.	
T2	0.80 (0.42 - 1.52)	0.505	0.97 (0.51 - 1.85)	0.938	1.21 (0.43 - 3.40)	0.722
T3	2.25 (1.36 - 3.74)	0.002	2.48 (1.49 - 4.13)	<0.001	2.85 (1.21 - 6.70)	0.016
MACE						
ACS						
SHR	1.56 (0.97 - 2.53)	0.069	1.83 (1.15 - 2.91)	0.011	1.73 (0.98 - 3.04)	0.058
T1	Ref.		Ref.		Ref.	
T2	0.85 (0.67 - 1.06)	0.153	0.92 (0.73 - 1.16)	0.480	0.96 (0.73 - 1.24)	0.740
T3	1.11 (0.89 - 1.39)	0.338	1.25 (1.00 - 1.56)	0.053	1.17 (0.90 - 1.52)	0.230
CCS						
SHR	1.85 (1.39 - 2.45)	<0.001	1.78 (1.35 - 2.34)	<0.001	1.69 (1.10 - 2.59)	0.017
T1	Ref.		Ref.		Ref.	
T2	0.78 (0.65 - 0.94)	0.008	0.88 (0.73 - 1.05)	0.155	0.97 (0.76 - 1.25)	0.839
T3	1.12 (0.95 - 1.32)	0.177	1.21 (1.03 - 1.43)	0.021	1.23 (0.96 - 1.56)	0.097

Model 1: univariate model; Model 2: adjust for age, and sex; Model 3: adjust for age, sex, BMI, SBP, smoking, drinking, TC, LDL-c, eGFR, and HR.

SHR, stress hyperglycemia ratio; CVD, cardiovascular disease; MACE, major adverse cardiovascular events; ACS, acute coronary syndrome; CCS, chronic coronary syndrome; HR,hazard ratio.

Besides, in the subgroup analysis of glucose status, SHR showed the stronger association with all-cause death, CVD death, and MACE in participants with DM. The association with CVD death in NGR could not be fully evaluated due to limited events (see **Table 4**).

**Table 4:** Association of SHR with all-cause death, CVD death, and MACE by glucose status.

	Model 1		Model 2		Model 3	
	HR (95%CI)	P value	HR (95%CI)	P value	HR (95%CI)	P value
All death						
NGR						
SHR	14.38 (4.26 - 48.55)	<0.001	12.52 (3.36 - 46.66)	<0.001	1.43 (0.12 - 17.70)	0.780
T1						
T2	0.37 (0.14 - 0.99)	0.047	0.63 (0.23 - 1.72)	0.368	0.46 (0.12 - 1.83)	0.271
T3	0.79 (0.33 - 1.91)	0.607	1.34 (0.55 - 3.23)	0.517	0.85 (0.24 - 2.93)	0.791
Pre-DM						
SHR	3.97 (1.62 - 9.70)	0.003	4.19 (1.73 - 10.11)	0.001	2.50 (0.55 - 11.43)	0.238
T1	Ref.		Ref.		Ref.	
T2	0.76 (0.48 - 1.19)	0.228	0.92 (0.59 - 1.45)	0.734	1.22 (0.67 - 2.21)	0.521
T3	1.37 (0.87 - 2.15)	0.171	1.55 (0.99 - 2.44)	0.057	1.60 (0.81 - 3.14)	0.173
DM						
SHR	2.54 (1.83 - 3.52)	<0.001	2.41 (1.73 - 3.34)	<0.001	2.51 (1.52 - 4.15)	<0.001
T1	Ref.		Ref.		Ref.	
T2	0.89 (0.67 - 1.18)	0.423	0.95 (0.71 - 1.26)	0.717	1.00 (0.66 - 1.51)	0.994
T3	1.52 (1.21 - 1.91)	<0.001	1.56 (1.24 - 1.97)	<0.001	1.50 (1.07 - 2.11)	0.019
CVD death						
NGR						
SHR	54.06 (11.78 - 248.2)	<0.001	93.44 (14.62 - 597.4)	<0.001	–	–
T1	Ref.		Ref.		Ref.	
T2	–	–	–	–	–	–
T3	–	–	–	–	–	–
Pre-DM						
SHR	6.14 (1.71 - 22.08)	0.005	4.30 (1.23 - 15.05)	0.022	6.28 (0.80 - 49.35)	0.081
T1	Ref.		Ref.		Ref.	
T2	0.74 (0.29 - 1.86)	0.518	0.89 (0.35 - 2.24)	0.801	1.20 (0.36 - 4.00)	0.771
T3	2.34 (1.03 - 5.29)	0.042	2.56 (1.13 - 5.83)	0.025	2.67 (0.81 - 8.82)	0.106
DM						
SHR	3.53 (1.95 - 6.38)	<0.001	3.26 (1.79 - 5.93)	<0.001	2.75 (1.04 - 7.28)	0.042
T1	Ref.		Ref.		Ref.	
T2	0.69 (0.36 - 1.34)	0.276	0.74 (0.38 - 1.42)	0.362	0.75 (0.30 - 1.84)	0.523
T3	2.11 (1.33 - 3.33)	0.001	2.14 (1.35 - 3.39)	0.001	1.97 (1.03 - 3.80)	0.042
MACE						
NGR						
SHR	4.64 (1.79 - 12.04)	0.002	5.04 (1.94 - 13.10)	0.001	1.70 (0.42 - 6.79)	0.455
T1	Ref.		Ref.		Ref.	
T2	0.79 (0.41 - 1.52)	0.473	0.98 (0.50 - 1.89)	0.942	0.90 (0.39 - 2.06)	0.803
T3	0.97 (0.52 - 1.84)	0.937	1.27 (0.67 - 2.41)	0.463	0.99 (0.44 - 2.21)	0.974
Pre-DM						
SHR	1.60 (0.80 - 3.18)	0.183	1.86 (0.96 - 3.60)	0.065	1.60 (0.80 - 3.18)	0.183
T1	Ref.		Ref.		Ref.	
T2	0.85 (0.65 - 1.09)	0.198	0.96 (0.75 - 1.25)	0.778	1.12 (0.83 - 1.53)	0.455
T3	0.98 (0.74 - 1.31)	0.911	1.10 (0.83 - 1.47)	0.516	1.12 (0.77 - 1.62)	0.550
DM						
SHR	1.68 (1.29 - 2.18)	<0.001	1.63 (1.25 - 2.11)	<0.001	1.68 (1.29 - 2.18)	<0.001
T1	Ref.		Ref.		Ref.	
T2	0.87 (0.72 - 1.05)	0.145	0.91 (0.75 - 1.09)	0.305	0.95 (0.75 - 1.22)	0.711
T3	1.23 (1.05 - 1.44)	0.011	1.27 (1.08 - 1.48)	0.003	1.26 (1.01 - 1.56)	0.038

Model 1: univariate model; Model 2: adjust for age, and sex; Model 3: adjust for age, sex, BMI, SBP, smoking, drinking, ACS, TC, LDL-c, eGFR, and HR.

SHR, stress hyperglycemia ratio; CVD, cardiovascular disease; MACE, major adverse cardiovascular events; NGR, normal glucose regulation; pre-DM, prediabetes mellitus; DM, diabetes mellitus; HR,hazard ratio.

## 4 Discussion

In this study, we explored the association between the SHR and long-term clinical outcomes of CAD, including all-cause death, CVD death, and MACE, in a large cohort of CAD during a median follow-up of 28 months. Our findings suggest that SHR was significantly associated with increased risks of long-term all-cause death, CVD death, and MACE. The RCS analysis further confirmed a positive nonlinear association between SHR and these adverse outcomes (all-cause death, CVD death, and MACE) and exhibited a J-shaped curve. The Kaplan-Meier curves revealed that the highest tertile (T3) group had the highest risk of all-cause death, CVD death, and MACE, while the lowest tertile (T1) group had the lowest risk (all log-rank P < 0.05). Further analysis using Cox proportional hazards regression showed that the T3 group had a significantly higher risk compared to the reference group (P < 0.05), while the differences between T2 and the reference group were not statistically significant. This indicates a clear risk gradient, with T3 having the highest risk and T1 having the lowest risk, suggesting that it has good stratification ability for identifying high-risk groups. Integrated with our previous study, the combined findings demonstrate that SHR is not only significantly associated with the risk of in-hospital mortality but also serves as an effective marker for risk stratification of long-term prognosis in patients with CAD (6).

Under the impact of emergency and critical illnesses such as severe trauma, infection, myocardial infarction, and stroke, the stress response leads to increased secretion of glucagon, adrenaline, and inflammatory factors, resulting in insulin resistance and ultimately causing an elevation in blood glucose levels (3, 8–10). As an important indicator of stress hyperglycemia, previous studies have sufficiently validated the relationship between the SHR and adverse prognosis in critical illnesses (11–14). Research on SHR in CAD has predominantly focused on patients with ACS or with limited follow-up duration, few studies focused on the entire CAD population both including ACS and CCS (5, 15–18). Yang et al. conducted a retrospective study for ACS patients who underwent drug-eluting stent and found that SHR presented U-shaped or J-shaped associations with early and late cardiovascular outcomes (5). Luo et al. enrolled 2,089 patients with acute myocardial infarction (AMI) and demonstrated that SHR can serve as a predictor of worse prognosis and may enhance the Global Registry of Acute Coronary Events (GRACE) score (16). During the median follow-up of 28 months, our study indicated a strong positive relationship between SHR and all-cause death, CVD death, and MACE among the CAD patients, with an approximately two-fold increased risk of all-cause death observed in the highest tertile (T3) compared to the lowest tertile (T1) in the fully adjusted model. Patients in T3 exhibited a significantly higher risk of all-cause death, CVD death, and MACE, compared to those in T1. These findings are consistent with prior research, highlighting the predictive value of SHR in worsening prognosis among patients with CAD.

### 4.1 Subgroup differences in SHR prognostic value

Subgroup analyses in this current study revealed a stronger association between SHR and long-term adverse outcomes (all-cause death, CVD death, and MACE) in patients with CCS compared to those with ACS. However, in our last study, we observed that SHR was significantly associated with in-hospital mortality in both ACS and CCS (6). The subgroup analyses from our two studies indicated that SHR held significant value in forecasting short-term prognosis in both ACS and CCS. However, its correlation with long-term prognosis appears to be more pronounced in CCS. This discrepancy likely stemmed from the different pathophysiological mechanisms underlying ACS and CCS. In CCS, chronic metabolic dysregulation and endothelial dysfunction may amplify the impact of stress hyperglycemia on long-term outcomes. Conversely, in ACS, the acute stress response gradually subsides after discharge, and chronic disease states may have a more significant impact on prognosis (19). The influence of stress hyperglycemia at admission on long-term prognosis may gradually diminish. In previously published literature, Schmitz et al. observed that stress hyperglycemia primarily had a significant impact on the short-term prognosis of patients with AMI, but barely had influence on long-term prognosis, validating the hypothesis that stress hyperglycemia affects poor prognosis through transient dynamic disorder when the acute event occurs (20). Additionally, the smaller scale of the ACS population in this study compared to the CCS group may have reduced statistical efficiency.

Furthermore, SHR exhibited a stronger predictive value for long-term adverse outcomes among individuals with DM in the subgroup analyses in this study. However, in our last study, SHR presented superior predictive power for in-hospital mortality in both the DM and pre-DM subgroups. In patients with DM, long-term blood glucose control is often difficult, and blood sugar levels may not return to normal even after an acute stress event. In contrast, patients with pre-DM have relatively lower glucose sugar levels and may more easily achieve normal levels through lifestyle and medication, which may lessen SHR’s impact on long-term prognosis. This underscores that in CAD patients with DM, those with higher SHR have both elevated short-term and long-term risks of adverse outcomes and require more attention, such as stricter blood glucose management. Besides, the limited number of CVD-related deaths in the NGR subgroup constrained our ability to fully assess this relationship. This finding aligns with prior studies suggesting that acute glucose fluctuations in DM may have detrimental cardiovascular effects beyond those observed in patients with chronic hyperglycemia.

### 4.2 Clinical implications and future directions

Our findings highlight the importance of SHR as a potential prognostic biomarker in CAD patients for predicting long-term outcomes, particularly those with CCS or DM. Given the increasing recognition of stress hyperglycemia as a cardiovascular risk factor, routine assessment of SHR in clinical practice may help identify high-risk individuals. Future studies should aim to validate our findings in larger, multi-center cohorts and explore the mechanistic pathways linking SHR to cardiovascular outcomes.

### 4.3 Limitations

The strengths of our study include a large sample size of CAD population and this study is an extension of our previous research on SHR and in-hospital mortality in CAD. We completed a median follow-up of 28 months, further exploring the impact of SHR on the long-term prognosis of CAD patients and conducted comprehensive analyses. However, several limitations should be acknowledged. First, the observational nature of this observational study may preclude causal inference. Second, glucose measurements were obtained at a single time point upon hospital admission, which may not fully capture dynamic glycemic changes over time. Third, the small number of CVD deaths in certain subgroups limited statistical power for subgroup analyses.

## 5 Conclusion

In conclusion, our study demonstrates that elevated SHR was significantly associated with increased risks of all-cause death, CVD death, and MACE in CAD patients during a median follow-up of 28 months, with stronger associations observed in those with CCS or DM. Our findings suggest that SHR may serve as a valuable marker of risk stratification for long-term prognosis in patients with CAD.

## Data Availability

The raw data supporting the conclusions of this article will be made available by the authors, without undue reservation.
